# Comparative efficacy of low volume versus traditional standard volume PEG on bowel preparation before colonoscopy: Protocol for an updated meta-analysis with trial sequential analysis

**DOI:** 10.1097/MD.0000000000010599

**Published:** 2018-04-27

**Authors:** Li-Juan Yi, Xu Tian, Yuan-Ping Pi, Ling Feng, Hui Chen, Xiao-Ling Liu, Wei-Qing Chen

**Affiliations:** a Department of Nursing, Hunan Traditional Chinese Medical College, Zhuzhou; b Department of Gastroenterology, Chongqing Key Laboratory of Translational Research for Cancer Metastasis and Individualized Treatment, Chongqing University Cancer Hospital and Chongqing Cancer Institute and Chongqing Cancer Hospital, Chongqing; c Editorial Office, TMR Integrative Nursing, TMR Publishing Group, Tianjin; d Department of Nursing, Key Laboratory for Biorheological Science and Technology of Ministry of Education (Chongqing University), Chongqing University Cancer Hospital and Chongqing Cancer Institute and Chongqing Cancer Hospital, Chongqing; e Department of Foundation Medicine, Hunan Traditional Chinese Medical College, Zhuzhou, China.

**Keywords:** bowel preparation, colonoscopy, meta-analysis, polyethylene glycol, trial sequential analysis

## Abstract

**Introduction:**

Polyethylene glycol (PEG) has been considered as the first recommendation for bowel preparation prior to colonoscopy. A previous meta-analysis suggested that low volume PEG may improve the acceptability of ingesting bowel preparation solution. However, several limitations impaired the power of findings from this published meta-analysis, such as the variation in study design of included trials and adjuvant prescriptions. Moreover, some studies related to this topic have been published recently. And thus, the aim of this updated meta-analysis is to further assess the comparative efficacy of low volume versus standard volume of PEG on bowel preparation before colonoscopy with trial sequential analysis (TSA).

**Methods and analysis:**

Systematic searches will be performed to capture any potential randomized controlled trials (RCTs) investigated the comparative efficacy of low volume versus traditional standard volume PEG on bowel preparation prior to colonoscopy in PubMed, EMBASE, and Cochrane Central Register of Controlled Trials. Moreover, we will also manually check the bibliographies of related studies and reviews so as to get additional studies. Two reviewers will independently screen the citation records, extract essential information, and appraise the risk of bias of each RCT in sequence. Finally, we will used the STATA software version 12.0 and TSA software version beta 0.9 to statistically analyze all data and test the robust of each pooled result, respectively.

**Results:**

We will submit the full-text of systematic review to a peer-review journal for publication.

**Conclusion:**

This updated systematic review and meta-analysis with TSA will further assess the comparative efficacy and safety of low-volume versus traditional standard volume PEG for bowel preparation prior to colonoscopy. And then, a more comprehensive evidence body on low-volume compared to standard volume PEG in bowel preparation will be constructed.

## Abstract 2

Supplemental Digital Content is available in the text

**Strengths and limitations of this study**

- The systematic review and meta-analysis addresses a critical question whether low volume PEG plus ascorbic acid compared to traditional standard volume should be recommended as a preferred option for the bowel preparation before colonoscopy.
- The systematic review and meta-analysis has a clearly defined aim and a strict inclusion criterion. Meanwhile, it describes how to screen citation records, extract data, assessment of the risk of bias, quantitative synthesis, and trial sequential analysis.
- The present systematic review and meta-analysis will design a table to comprehensively document all results from the published and the present meta-analyses.
- A series of established methods will be designed to improve the reliability of the pooled results through rationally addressing heterogeneity and the risk of bias.
- Limitations include the variation in directions (eg, split or single, morning, or afternoon colonoscopy) and tools of assessing bowel preparation efficacy, which may affect the comparison results.

## 1 Introduction

Colonoscopy has been deemed to be the critical method of early diagnosing lesions in digestive tract, screening colorectal cancer as well as invasive treatment.^[1,2]^ Nevertheless, it must be noted that the efficacy and safety of colonoscopy are mainly dependent upon adequate bowel preparation and patient attendance.^[3–5]^ In practice, large volume of preparation solutions will be administered to patients who are assigned to receive colonoscopy. However, it is estimated that approximately 25% to 33% patients failed to achieve the adequate bowel preparations, because the patients are intolerant to volume-related discomfort.^[6,7]^ Published evidences suggested that inadequate bowel preparation are associated with lower rates of cecal intubation,^[8]^ higher operational difficulty,^[9]^ lower adenoma detection rates, and greater financial costs.^[10–12]^

Polyethylene glycol (PEG) remains the first recommended regimen for bowel cleansing prior to colonoscopy.^[13,14]^ However, in order to obtain adequate bowel cleaning, patients will be instructed to digest 4 L PEG, and thus the acceptance and compliance with this given regime will be weakened.^[15,16]^ In addition, these limitations also decreased the courage of patients to participate in the regular colonoscopy surveillance.^[17,18]^ Considering the limitations of traditional high volume PEG regime, researchers and practitioners turned attention to modified options, and several studies have found that low volume PEG combined with ascorbic acid (Asc) may have the potential of addressing the issues faced by traditional PEG regime.^[19–21]^ Asc is helpful because it can allow halving the volume of the lavage solution without the loss of efficacy and disgusting taste.^[22,23]^ Several randomized controlled trials (RCTs)^[24–26]^ have consistently shown that low volume PEG plus Asc regime achieved similar efficacy compared to standard volume one. Similarly, the findings from a previous meta-analysis^[27]^ is in accordance with aforementioned studies. However, this meta-analysis considered a quasi-randomized trial^[28]^ and ignored the variation in adjuvants (Bisacodyl and Simethicone),^[29,30]^ which potentially damaged the power of summary results.

Considering the above information, we designed this updated meta-analysis to comprehensively investigate the comparative efficacy of low volume PEG plus Asc related to traditional volume PEG alone for bowel preparation before colonoscopy. In order to test whether a conclusive conclusion for a specific outcome can be drawn, we will also perform trial sequential analysis to calculate the accumulated sample size and required information size associated with all outcomes in our study. We designed this systematic review on January 20, 2018, and we expected to complete this study on May 20, 2018.

## 2 Methods and design

**Figure 1:**
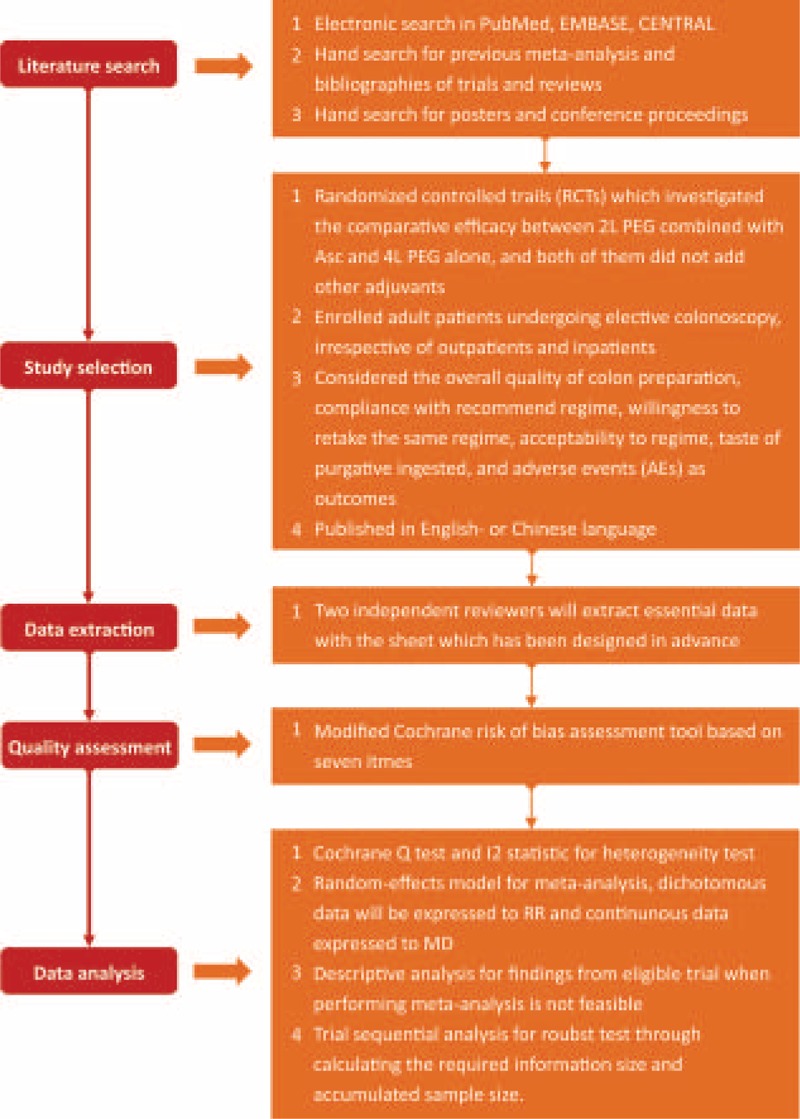
Flow chart of this updated systematic review and meta-analysis. AE =adverse event, Asc = ascorbic acid, CENTRAL = Cochrane Central Register of Controlled Trials, MD = mean difference, PEG = polyethylene glycol, RCT = randomized controlled trial, RR = relative risk, TSA = trial sequential analysis.

We designed and finished this protocol for a meta-analysis in line with the Preferred Reporting Items for Systematic Reviews and meta-analysis protocols 2015: elaboration and explanation.^[31]^ This protocol has been registered in International Prospective Register of Systematic Reviews and a register number of CRD42018089827 was approved. We will carry out the full systematic review and meta-analysis in consistent with the recommendations proposed by Cochrane Collaboration.^[32]^ What is more, all results will be reported according to the Preferred Reporting Items for Systematic Reviews and meta-analysis statement.^[31]^ The written informed consent will not be needed, because all analyses will be completed based on published data. The Fig. 1 shows the flow chat of this ipdated systematic review and meta-analysis.

### 2.1 Selection criteria

We prespecified the inclusion criteria. Study will be considered if the following criteria are met: all adult patients undergoing elective colonoscopy, irrespective of outpatients, and inpatients; RCTs which investigated the comparative efficacy between 2L PEG combined with Asc and 4L PEG alone were considered, and both of them did not add other adjuvants; the overall quality of colon preparation was regarded as primary outcome, and the secondary outcomes included compliance with recommend regime, willingness to retake the same regime, acceptability to regime, taste of purgative ingested, and adverse events; and only studies published in English and Chinese will be permitted.

Article will be excluded if it met at least one of the following criteria: essential information which cannot be extracted and obtained from authors; duplicates (derive from the same research group) with poor methodology and insufficient data.

### 2.2 Definition of outcomes

The overall quality of colon preparation was predefined as successful bowel cleansing in our study. For the purposes of the analysis, the successful preparation was reached when conformed to one of following conditions: an Ottawa score of <5; a Boston Bowel Preparation Scale score of ≥2 for all segments; a grade of either excellent or good on the Aronchik scale; grades A and B according to the Harefield Cleansing Scale; and other nonvalidated 3-, 4-, or 5-point scales (excellent, good, fair, poor, and very poor).

Compliance to the regimen was assessed by asking the patients how much the dosing regimen they have ingested. We predefined good compliance as consumption of ≥75% of the regime and excellent compliance as consumption of 100% of the regime. In terms of subjective indexes, willingness to retake the same regime, acceptability to regime, and taste of purgative ingested were measured by using an unofficial questionnaire in each suitable study (ie, produced by individual study). All adverse events related to bowel preparation were monitored and recorded during colonoscopy.

### 2.3 Identification of citations

An electronic search will be performed to collect any potential RCTs investigating the comparative efficacy of 2 target PEG-based regimes in PubMed, EMBASE, and Cochrane Central Register of Controlled Trials from January 2000 to April 2018. Search results will be updated weekly in order to timely capture any recent studies. “Colonoscopy,” “polyethylene glycols,” and “random” will be used to construct search strings based on medical subject heading and free word which are embedded in specific files involving title, keywords, and abstract. All search algorithms have been designed for targeted databases, and we have documented these algorithms in Supplemental Table 1, http://links.lww.com/MD/C225.

In addition, we will also replenish the potential studies through manually checking the bibliographies of eligible studies and relevant reviews. Two reviewers will independently and critically examine citations by reading the titles, abstracts, and full-texts in turn.

### 2.4 Data extraction

A predesigned data extraction form was designed by the review authors. All acquired citations will be imported into EndNote software. Whereafter, 2 reviewers will independently extract the essential information, in which involves first author, publication year, risk of bias, age of participants, sample size (male/female), bowel preparation scale, the description of intervention (study group/control group), dietary instruction, and outcomes of interest. We will contact the leading authors of relevant articles in case extractive data are not available. All information will be rechecked mutually. We will calculate the Kappa value to assess the interinvestigator reliability. We will also organize the consensus principles which serve as the means of resolving divergences among reviewers.

### 2.5 Quality assessment

Two reviewers will independently adopt the modified tool based on Cochrane tool to appraise the risk of bias^[32,33]^ of each included study. The risk of bias will be assessed from 8 domains severally, including randomization sequence generation, allocation concealment, blinding of participants, blinding of study personnel, blinding of outcome assessors, incomplete outcome data, selective reporting, and other bias. Besides, the evaluation results will be cross-checked. The risk of each domain will be rated as “high risk of bias,” “unclear risk of bias,” and “low risk of bias” according to the match level between extractive information and evaluation criteria. Any disagreements will be resolved by discussing with a 3rd senior reviewer.

### 2.6 Statistical analysis

We will input all extracted data to STATA software version 12.0 (Stata Corp, College Station, TX) for statistical analyses. The estimates of dichotomous data will be expressed as relative risk and 95% confidence intervals. The estimates of continuous data will be expressed as mean differences or standard mean differences with 95% confidence intervals. Heterogeneity in included studies will be qualitatively evaluated using the Cochrane Q, and the proportion of overall variation that is attributable to between-study heterogeneity will be quantitatively evaluated by *I*^2^ statistic.^[34,35]^

We will analyze the clinical diversity and methodological comparability of every suitable study firstly according to the characteristics of the participants, research design and method, intervention regimes, and measurement and statistical analysis of outcomes. If the clinical characteristic and methodology are considered heterogeneity, qualitative analysis will be used. If not, we will use the Cochrane Q to qualitatively evaluate the heterogeneity in studies in terms of each outcome.^[36]^ Moreover, the level of heterogeneity will be quantified by the *I*^2^ statistic. If *I*^2^ is <50%, the suitable studies will be considered to be homogeneous; in contrast, the pooled results will be affected by substantial heterogeneity. We adopted random-effect model based on Mantel–Haenszel or inverse variance approach to perform all analyses. As to the compliance with recommend regime, subgroup analyses will be planned according to the total consumption of the regime. If the number of studies analyzed in single outcome is more than 10, publication bias will be detected by using Egger test.^[37,38]^ If study with multiple-arm design is included, we will extract the data from intervention groups which are up to the inclusion criteria according to the recommendations proposed by Cochrane Collaboration.^[32]^

## 3 Discussion

Subjects’ participation and adequate bowel cleansing are the essential requirements for a high-quality colonoscopy.^[3,28,39]^ Therefore, the ideal colon cleansing should be capable of emptying the colon of all fecal material without damaging its mucosa, causing no discomfort, and minimizing fluids and electrolyte imbalance.^[40,41]^ Traditional 4 L PEG regime has been used worldwide for its high efficacy, lower price, and superior safety.^[42,43]^ But volume-related discomfort and unpleasant taste may deter the acceptability with colonoscopy. It is closely linked to subject’ s attendance.^[44]^ Poor acceptability will impair the willingness to take the examination in the future.^[17,18]^ Recently, low-volume PEG regime shows a better toleration under the condition that its cleanliness is equivalent to that of traditional 4 L PEG regimen.^[19,24,25,45]^ Excessive Asc that remains in the bowel exerts an osmotic effect,^[22,23]^ thereby, it reduces the quantity of PEG.^[44]^

Although a previous meta-analysis has reported that the efficacy of low-volume PEG plus Asc is comparable with that of traditional 4 L PEG regime, several limitations (such as the diversity in study designs and adjuvant prescriptions) deterred the reliability of pooled results. Moreover, some potential RCTs have been published recently. In order to further determine the comparative role between low volume PEG regime and traditional large volume one in bowel preparation prior to colonoscopy, we designed this updated systematic review and meta-analysis for the purpose of facilitating the decision making. Meanwhile, we also plan to use trial sequential analysis technique to calculate the accumulated sample size of each outcome in order to determine the robust of findings.

## Acknowledgments

The authors thank the coauthors and all authors, who have contributed significantly.

## Author contributions

**Data acquisition:** Li-Juan Yi, Xu Tian.

**Drafted the manuscript, which was critically revised for intellectual content by Xu Tian and Wei-Qing Chen:** Li-Juan Yi, Ling Feng, Hui Chen.

**Interpreted the data and results of the analyses:** Li-Juan Yi, Xu Tian, Yuan-Ping Pi, Xiao-Ling Liu.

**Performed statistical analysis:** Li-Juan Yi, Xu Tian, Hui Chen.

**Study conception and design:** Li-Juan Yi, Xu Tian, Yuan-Ping Pi, Ling Feng, Wei-Qing Chen.

All authors read and approved the final manuscript.

**Conceptualization:** Li-juan Yi, Xu Tian, Yuan-Ping Pi, Ling Feng, Wei-Qing Chen.

**Data curation:** Yuan-Ping Pi, Ling Feng, Xiao-ling Liu.

**Formal analysis:** Li-juan Yi, Xu Tian, Hui Chen, Xiao-ling Liu, Wei-Qing Chen.

**Methodology:** Li-juan Yi, Xu Tian, Wei-Qing Chen.

**Supervision:** Li-juan Yi.

**Writing – original draft:** Li-juan Yi, Ling Feng, Hui Chen.

**Writing – review & editing:** Xu Tian, Yuan-Ping Pi.

## Supplementary Material

Supplemental Digital Content

## References

[1] Gentile M De Rosa M Cestaro G 2 L PEG plus ascorbic acid versus 4 L PEG plus simethicon for colonoscopy preparation: a randomized single-blind clinical trial. Surg Laparosc Endosc Percutan Tech 2013;23:276–80. 23751992 10.1097/SLE.0b013e31828e389d

[2] West NJ Boustiere C Fischbach W Colorectal cancer screening in Europe: differences in approach; similar barriers to overcome. Int J Colorectal Dis 2009;24:731–40. 19296117 10.1007/s00384-009-0690-6

[3] Bernstein C Thorn M Monsees K A prospective study of factors that determine cecal intubation time at colonoscopy. Gastrointest Endosc 2005;61:72–5. 15672059 10.1016/s0016-5107(04)02461-7

[4] Seo JY Lee C Jin EH Is a split-dose regimen of 2 L polyethylene glycol plus ascorbic acid tolerable for colonoscopy in an early morning visit to a comprehensive medical check-up? World J Gastroenterol 2017;23:1030–7. 28246476 10.3748/wjg.v23.i6.1030 PMC5311091

[5] Rodriguez de Miguel C Serradesanferm A Lopez-Ceron M Ascorbic acid PEG-2L is superior for early morning colonoscopies in colorectal cancer screening programs: a prospective non-randomized controlled trial. Gastroenterol Hepatol 2015;38:62–70. 25458542 10.1016/j.gastrohep.2014.09.007

[6] Moon W Optimal and safe bowel preparation for colonoscopy. Clin Endosc 2013;46:219–23. 23767029 10.5946/ce.2013.46.3.219 PMC3678056

[7] Siddiqui AA Yang K Spechler SJ Duration of the interval between the completion of bowel preparation and the start of colonoscopy predicts bowel-preparation quality. Gastrointest Endosc 2009;69 (3 Pt 2):700–6. 19251013 10.1016/j.gie.2008.09.047

[8] Zuber-Jerger I Kullmann F A prospective study of factors that determine cecal intubation time at colonoscopy. Gastrointest Endosc 2006;63:358–9. 10.1016/j.gie.2005.09.007 16427963

[9] Valiante F Bellumat A De Bona M Bisacodyl plus split 2-L polyethylene glycol-citrate-simethicone improves quality of bowel preparation before screening colonoscopy. World J Gastroenterol 2013;19:5493–9. 24023492 10.3748/wjg.v19.i33.5493 PMC3761102

[10] Lebwohl B Kastrinos F Glick M The impact of suboptimal bowel preparation on adenoma miss rates and the factors associated with early repeat colonoscopy. Gastrointest Endosc 2011;73:1207–14. 21481857 10.1016/j.gie.2011.01.051 PMC3106145

[11] Chokshi RV Hovis CE Hollander T Prevalence of missed adenomas in patients with inadequate bowel preparation on screening colonoscopy. Gastrointest Endosc 2012;75:1197–203. 22381531 10.1016/j.gie.2012.01.005

[12] Rex DK Imperiale TF Latinovich DR Impact of bowel preparation on efficiency and cost of colonoscopy. Am J Gastroenterol 2002;97:1696–700. 12135020 10.1111/j.1572-0241.2002.05827.x

[13] Yoo IK Jeen YT Kang SH Improving of bowel cleansing effect for polyethylene glycol with ascorbic acid using simethicone: a randomized controlled trial. Medicine 2016;95:e4163. 27428209 10.1097/MD.0000000000004163 PMC4956803

[14] Saltzman JR Cash BD Pasha SF Bowel preparation before colonoscopy. Gastrointest Endosc 2015;81:781–94. 25595062 10.1016/j.gie.2014.09.048

[15] Radaelli F Meucci G Sgroi G Technical performance of colonoscopy: the key role of sedation/analgesia and other quality indicators. Am J Gastroenterol 2008;103:1122–30. 18445096 10.1111/j.1572-0241.2007.01778.x

[16] Valiante F Pontone S Hassan C A randomized controlled trial evaluating a new 2-L PEG solution plus ascorbic acid vs 4-L PEG for bowel cleansing prior to colonoscopy. Dig Liver Dis 2012;44:224–7. 22119219 10.1016/j.dld.2011.10.007

[17] Tan JJ Tjandra JJ Which is the optimal bowel preparation for colonoscopy – a meta-analysis. Colorectal Dis 2006;8:247–58. 16630226 10.1111/j.1463-1318.2006.00970.x

[18] Friedman S Cheifetz AS Farraye FA Factors that affect adherence to surveillance colonoscopy in patients with inflammatory bowel disease. Inflamm Bowel Dis 2013;19:534–9. 23429444 10.1097/MIB.0b013e3182802a3c

[19] Bitoun A Ponchon T Barthet M Results of a prospective randomised multicentre controlled trial comparing a new 2-L ascorbic acid plus polyethylene glycol and electrolyte solution vs. sodium phosphate solution in patients undergoing elective colonoscopy. Aliment Pharmacol Ther 2006;24:1631–42. 17094774 10.1111/j.1365-2036.2006.03167.x

[20] Mamula P Adler DG Conway JD Colonoscopy preparation. Gastrointest Endosc 2009;69:1201–9. 19481646 10.1016/j.gie.2009.01.035

[21] Norgine Ltd. Data on File. Harefield,UK: Norgine. 2006.

[22] Wilson JX Regulation of vitamin C transport. Ann Rev Nutr 2005;25:105–25. 16011461 10.1146/annurev.nutr.25.050304.092647

[23] Fujita I Akagi Y Hirano J Distinct mechanisms of transport of ascorbic acid and dehydroascorbic acid in intestinal epithelial cells (IEC-6). Res Commun Mol Pathol Pharmacol 2000;107:219–31. 11484876

[24] Moon CM Park DI Choe YG Randomized trial of 2-L polyethylene glycol + ascorbic acid versus 4-L polyethylene glycol as bowel cleansing for colonoscopy in an optimal setting. J Gastroenterol Hepatol 2014;29:1223–8. 24955451 10.1111/jgh.12521

[25] Ell C Fischbach W Bronisch HJ Randomized trial of low-volume PEG solution versus standard PEG + electrolytes for bowel cleansing before colonoscopy. Am J Gastroenterol 2008;103:883–93. 18190651 10.1111/j.1572-0241.2007.01708.x

[26] Jansen SV Goedhard JG Winkens B Preparation before colonoscopy: a randomized controlled trial comparing different regimes. Eur J Gastroenterol Hepatol 2011;23:897–902. 21900786 10.1097/MEG.0b013e32834a3444

[27] Xie Q Chen L Zhao F A meta-analysis of randomized controlled trials of low-volume polyethylene glycol plus ascorbic acid versus standard-volume polyethylene glycol solution as bowel preparations for colonoscopy. PloS One 2014;9:e99092. 24902028 10.1371/journal.pone.0099092 PMC4047058

[28] Corporaal S Kleibeuker JH Koornstra JJ Low-volume PEG plus ascorbic acid versus high-volume PEG as bowel preparation for colonoscopy. Scand J Gastroenterol 2010;45:1380–6. 20602568 10.3109/00365521003734158

[29] Parente F Vailati C Bargiggia S 2-Litre polyethylene glycol-citrate-simethicone plus bisacodyl versus 4-litre polyethylene glycol as preparation for colonoscopy in chronic constipation. Dig Liver Dis 2015;47:857–63. 26232311 10.1016/j.dld.2015.06.008

[30] Zhang S Zheng D Wang J Simethicone improves bowel cleansing with low-volume polyethylene glycol: a multicenter randomized trial. Endoscopy 2017;50:412–22. 29132175 10.1055/s-0043-121337

[31] Moher D Liberati A Tetzlaff J Preferred reporting items for systematic reviews and meta-analyses: the PRISMA statement. Int J Surg (Lond, Engl) 2010;8:336–41. 10.1016/j.ijsu.2010.02.007 20171303

[32] Higgins JP Green S Cochrane Handbook for Systematic Reviews of Interventions Version 5.0.0. Naunyn-Schmiedebergs Archiv für experimentelle Pathologie und Pharmakologie 2009;210:S38.

[33] Zeng X Zhang Y Kwong J The methodological quality assessment tools for preclinical and clinical studies, systematic review and meta-analysis, and clinical practice guideline: a systematic review. J Evid Based Med 2015;8:2–10. 25594108 10.1111/jebm.12141

[34] Higgins J Thompson S Quantifying heterogeneity in a meta-analysis. Stat Med 2002;21:1539–58. 12111919 10.1002/sim.1186

[35] Lu G Ades A Combination of direct and indirect evidence in mixed treatment comparisons. Stat Med 2004;23:3105–24. 15449338 10.1002/sim.1875

[36] Higgins J Thompson S Deeks J Measuring inconsistency in meta-analyses. BMJ 2003;327:557–60. 12958120 10.1136/bmj.327.7414.557 PMC192859

[37] Egger M Davey Smith G Schneider M Bias in meta-analysis detected by a simple, graphical test. BMJ 1997;315:629–34. 9310563 10.1136/bmj.315.7109.629 PMC2127453

[38] Sterne JAC Sutton AJ Ioannidis JPA Recommendations for examining and interpreting funnel plot asymmetry in meta-analyses of randomised controlled trials. BMJ 2011;343:d4002. 21784880 10.1136/bmj.d4002

[39] Hwang KL Chen WT Hsiao KH Prospective randomized comparison of oral sodium phosphate and polyethylene glycol lavage for colonoscopy preparation. World J Gastroenterol 2005;11:7486–93. 16437721 10.3748/wjg.v11.i47.7486 PMC4725166

[40] Nelson DB Barkun AN Block KP Technology Status Evaluation report. Colonoscopy preparations. May 2001. Gastroint Endosc 2001;54:829–32. 11726878

[41] Wexner SD Beck DE Baron TH A consensus document on bowel preparation before colonoscopy: prepared by a task force from the American Society of Colon and Rectal Surgeons (ASCRS), the American Society for Gastrointestinal Endoscopy (ASGE), and the Society of American Gastrointestinal and Endoscopic Surgeons (SAGES). Dis Colon Rectum 2006;49:792–809. 16741637 10.1007/s10350-006-0536-z

[42] Hassan C Bretthauer M Kaminski MF Bowel preparation for colonoscopy: European Society of Gastrointestinal Endoscopy (ESGE) guideline. Endoscopy 2013;45:142–50. 23335011 10.1055/s-0032-1326186

[43] Mathus-Vliegen E Pellise M Heresbach D Consensus guidelines for the use of bowel preparation prior to colonic diagnostic procedures: colonoscopy and small bowel video capsule endoscopy. Curr Med Res Opin 2013;29:931–45. 23659560 10.1185/03007995.2013.803055

[44] Senore C Ederle A Fantin A Acceptability and side-effects of colonoscopy and sigmoidoscopy in a screening setting. J Med Screen 2011;18:128–34. 22045821 10.1258/jms.2011.010135

[45] Ponchon T Boustiere C Heresbach D A low-volume polyethylene glycol plus ascorbate solution for bowel cleansing prior to colonoscopy: the NORMO randomised clinical trial. Dig Liver Dis 2013;45:820–6. 23769755 10.1016/j.dld.2013.04.009

