# Supplementary material for: Comparative efficacy of low volume versus traditional standard volume PEG on bowel preparation before colonoscopy: Protocol for an updated meta-analysis with trial sequential analysis
Source: Medicine (Baltimore). 2018 Apr 27;97(17):e0599. doi: 10.1097/MD.0000000000010599 (PMC5944532; doi:10.1097/MD.0000000000010599)
Supplement: Supplemental Digital Content [file medi-97-e0599-s001.pdf]

**Recent queries in pubmed**	
Search	Query
#8	#3 AND #6 AND #7
#7	Search random* Sort by: PublicationDate
#6	#4 OR #5
#5	Search (((((((Colonoscop*) OR Colonoscopic Surgical Procedure*) OR Procedure, Colonoscopic Surgical) OR Procedures, Colonoscopic Surgical) OR Surgical Procedure, Colonoscopic) OR Surgery, Colonoscopic) OR Surgical Procedures, Colonoscopic) OR Colonoscopic Surger*) OR Surgeries, Colonoscopic Sort by: PublicationDate
#4	Search "Colonoscopy"[Mesh] Sort by: PublicationDate
#3	#1 OR #2
#2	Search ((((((((Polyethylene Glycol*) OR Macrogol*) OR Glycol, Polyethylene) OR Glycols, Polyethylene) OR Polyethylene Oxide*) OR Oxide, Polyethylene) OR Oxides, Polyethylene) OR Polyethyleneoxide*) OR Polyoxyethylene*) OR Tritons Sort by: PublicationDate
#1	Search "Polyethylene Glycols"[Mesh] Sort by: PublicationDate

Recent queries in CENTRAL	
Search	Query
#8	#3 AND #6 AND #7
#7	random*:ti,ab,kw(Word variations have been searched)
#6	#4 OR #5
#5	Colonoscop*:ti,ab,kw OR Colonoscopic Surgical Procedure*:ti,ab,kw OR ‘Procedure, Colonoscopic Surgical’:ti,ab,kw OR ‘Procedures, Colonoscopic Surgical’:ti,ab,kw OR ‘Surgical Procedure, Colonoscopic’:ti,ab,kw OR ‘Surgery, Colonoscopic’:ti,ab,kw OR ‘Surgical Procedures, Colonoscopic’:ti,ab,kw OR Colonoscopic Surger*:ti,ab,kw OR ‘Surgeries, Colonoscopic’:ti,ab,kw(Word variations have been searched)
#4	MeSH descriptor: [Colonoscopy] explode all trees
#3	#1 OR #2
#2	Polyethylene Glycol*:ti,ab,kw OR Macrogol*:ti,ab,kw OR ‘Glycol, Polyethylene’:ti,ab,kw OR ‘Glycols, Polyethylene’:ti,ab,kw OR Polyethylene Oxide*:ti,ab,kw(Word variations have been searched)
#1	MeSH descriptor: [Polyethylene Glycols] explode all trees

Recent queries in Embase	
Search	Query
#10	#3 AND #6 AND #9
#9	#7 OR #8
#8	Search 'randomized controlled trial'/exp OR 'randomized controlled trial (topic)'/exp
#7	Search random*
#6	#4 OR #5
#5	Search colonoscop* OR (colonoscopic AND surgical AND procedure*) OR (procedure, AND colonoscopic AND surgical) OR (procedures, AND colonoscopic AND surgical) OR (surgical AND procedure, AND colonoscopic) OR (surgery, AND colonoscopic) OR (surgical AND procedures, AND colonoscopic) OR (colonoscopic AND surger*) OR (surgeries, AND colonoscopic)
#4	Search 'colonoscopy'/exp
#3	#1 OR #2
#2	Search polyethylene AND glycol* OR macrogol* OR (glycol, AND polyethylene) OR (glycols, AND polyethylene) OR (polyethylene AND oxide*) OR (oxide, AND polyethylene) OR (oxides, AND polyethylene) OR polyethyleneoxide* OR polyoxyethylene OR tritons
#1	Search 'macrogol derivative'/exp
